# Age and sex are associated with Alzheimer's disease neuropathology in Down syndrome

**DOI:** 10.1002/alz.70408

**Published:** 2025-07-17

**Authors:** Elizabeth J. Andrews, Phong T. Ngo, Jesse R. Pascual, Freddy Gonzalez, Michael Phelan, Sierra T. Wright, Jordan Harp, Frederick Schmitt, Florence Lai, Patrick J. Lao, Adam M. Brickman, Julia Kofler, Milos D. Ikonomovic, Elizabeth Head, for the ABC‐DS Consortium

**Affiliations:** ^1^ Department of Pathology & Laboratory Medicine 1111 Gillespie Neuroscience Research Facility University of California Irvine Irvine California USA; ^2^ Department of Neurology Sanders Brown Center on Aging University of Kentucky Lexington Kentucky USA; ^3^ Department of Neurology, Massachusetts General Hospital Harvard Medical School Charlestown Massachusetts USA; ^4^ Taub Institute for Research on Alzheimer's Disease and the Aging Brain Department of Neurology Columbia University New York New York USA; ^5^ Department of Pathology University of Pittsburgh School of Medicine Pittsburgh Pennsylvania USA; ^6^ Geriatric Research Education and Clinical Center VA Pittsburgh HS University Drive C Pittsburgh Pennsylvania USA; ^7^ Department of Neurology University of Pittsburgh School of Medicine Pittsburgh Pennsylvania USA; ^8^ Department of Psychiatry University of Pittsburgh School of Medicine Pittsburgh Pennsylvania USA

**Keywords:** Alzheimer disease progression, beta‐amyloid, neuropathology, tau, trisomy 21

## Abstract

**INTRODUCTION:**

This study investigates the association of age and biological sex with Alzheimer's disease (AD) neuropathology in Down syndrome (DS).

**METHODS:**

We examined the frontal/occipital cortex in people with DS (*n* = 14/13, 1–39 years), DS with AD (DSAD) neuropathology (*n* = 18/19, 42–61 years), late‐onset AD (*n* = 15/16, 72–96 years), and age‐matched controls (*n* = 50/47)(*n* = 156). The area occupied by AT8 and 6E10 immunolabeling, representing tangle and plaque loads, respectively, was used for segmented linear regression analyses.

**RESULTS:**

There was elevated neuropathology after age 35 in DSAD, with inflection points at ∼31 years (amyloid‐β [Aβ]) and ∼28 (phosphorylated tau [p‐tau]) in the frontal cortex and ∼36 years (both Aβ and p‐tau) in the occipital cortex. Occipital p‐tau was higher in women relative to men with DS. Aβ and p‐tau pathology were correlated in women with DS but not in men with DS in the occipital cortex.

**DISCUSSION:**

Women with DS may show a more advanced stage of tau pathology relative to men with DS.

**Highlights:**

- Amyloid‐β (Aβ) and phosphorylated tau (p‐tau) Alzheimer's disease (AD) pathology emerge after 30 years of age in the frontal cortex, followed 7 years later by pathology in the occipital cortex in Down syndrome (DS).
- Women with DS show a more rapid progression of AD neuropathology seen by trends in higher p‐tau relative to men, despite similar levels of Aβ.
- Women with DS show a stronger association between Aβ and tau in the occipital but not frontal cortex relative to men with DS, independent of age.

## 1 BACKGROUND

People with Down syndrome (DS) are disproportionately affected by Alzheimer's disease (AD), due to triplication of chromosome 21, which contains the amyloid precursor protein (APP) gene.^1^, ^2^ By the age of 40 years, virtually all people with full trisomy 21 exhibit AD pathology, marked by amyloid‐β (Aβ) plaques and neurofibrillary tangles (NFT, phosphorylated tau [p‐tau]).^3^, ^4^ Onset of dementia in people with DS typically occurs over 10 years after AD neuropathology accumulates, with the median age of onset at 55.5 years.^5^, ^6^ As the population of people with DS is now living longer, it becomes increasingly critical to understand the neurobiological mechanisms underlying AD.^7^

As in late‐onset AD, age is the strongest risk factor for developing AD in people with DS.^8^, ^9^ Aβ deposition can be observed as early as 12 years of age and is systematically present over the age of 30 years in DS.^10^ Deposition of AD pathology follows a similar trajectory as in late onset AD, although it occurs at much younger ages.^9^ In addition to age, there is increasing evidence of sex differences in AD pathology.^11^

In the neurotypical population, about two‐thirds of people with AD are women.^12^ Women with AD tend to have a higher pathologic burden, particularly p‐tau.^11^ Greater p‐tau burden in women is also observed with positron emission tomography (PET) imaging in individuals with preclinical AD, suggesting women are more vulnerable to p‐tau accumulation even before the onset of dementia.^11^, ^13^

However, in DS, it is unclear if there are sex differences in AD pathology or course.^14^ There are conflicting reports of sex differences in dementia risk and course in people with DS. For example, women with DS were more likely to die from dementia than men.^15^, ^16^ However, one study suggested that sex did not influence the prevalence of dementia at the time of assessment (defined by the authors as risk of dementia); women with DS showed a longer duration of dementia than men with DS.^17^ Men with DS over the age of 60 years were 6.32 times more likely to have dementia than women with DS of the same age.^18^ Neuropathology studies are limited, but in one study, women had a higher burden of neurofibrillary tau tangles, despite having similar levels of Aβ, as men.^19^

In this study, we hypothesized that, compared to men, women with DS would exhibit more p‐tau pathology at autopsy, with little to no differences seen in Aβ pathology. To test this hypothesis, we quantified the extent of Aβ and p‐tau pathology in frontal and occipital cortices using digital whole slide images (WSI) of *post mortem* human brain sections stained with Aβ (6E10) and p‐tau (AT8) antibodies. The frontal cortex is a site of earlier Aβ and p‐tau pathology deposition compared to the occipital cortex, which is typically affected later in the disease.^20^, ^21^ The frontal cortex is also involved in higher‐level cognitive functioning and is one of the key regions impacted in AD, making it critical to understand disease progression. By contrast, the occipital cortex tends to develop AD pathology later, yet it is preferentially affected by cerebrovascular pathology in DS, making it a vulnerable region in the progression of AD in DS.^22^

## 2 METHODS

### 2.1 Human *post mortem* tissue

Coronal sections of frontal (BA8‐9: FCX) and occipital cortex (BA17‐18: OCC) were taken from human *post mortem* brain tissue collected at autopsy from two repositories: University of California at Irvine (UCI) Alzheimer Disease Research Center (ADRC) and the National Institutes of Health (NIH) NeuroBiobank. All cases had *post mortem* intervals (PMI) of less than 24 h. See Table 1 for demographic information. Tissue was either fixed in 4% paraformaldehyde before being transferred to PBS with 0.02% sodium azide for long‐term storage at 4 degrees (UCI ADRC) or fixed in formalin and stored at room temperature (NIH Neurobiobank).

**TABLE 1:** *Post mortem* tissue data—frontal and occipital cortices.

Frontal cortex	YC *N* = 14*^1^*	DS *N* = 14*^1^*	MC *N* = 18*^1^*	DSAD *N* = 18*^1^*	AC *N* = 18*^1^*	AD *N* = 15*^1^*
Sex						
Female	7 (50%)	6 (43%)	8 (44%)	9 (50%)	10 (56%)	6 (40%)
Male	7 (50%)	8 (57%)	10 (56%)	9 (50%)	8 (44%)	9 (60%)
PMI, hours	12 (8, 17)	18 (12, 24)	16 (7, 20)	5 (4, 11)	4 (3, 5)	5 (4, 8)
Age, years	22 (16, 33)	28 (2, 40)	51 (45, 64)	52 (49, 56)	83 (78, 88)	83 (80, 86)
*^1^* *n* (%); Median (Q1, Q3)						

Occipital cortex	YC *N* = 12*^1^*	DS *N* = 13*^1^*	MC *N* = 18*^1^*	DSAD *N *= 19*^1^*	AC *N* = 17*^1^*	AD *N* = 16*^1^*
Sex						
Female	4 (33%)	5 (38%)	7 (39%)	10 (53%)	11 (65%)	7 (44%)
Male	8 (67%)	8 (62%)	11 (61%)	9 (47%)	6 (35%)	9 (56%)
PMI, hours	17 (12, 23)	18 (14, 24)	15 (6, 20)	6 (5, 18)	5 (3, 9)	6 (4, 10)
Age, years	22 (16, 25)	25 (19, 39)	54 (46, 64)	54 (49, 56)	81 (78, 89)	81 (78, 86)
*^1^* *n* (%); Median (Q1, Q3)						

Abbreviations: AC, aged control; AD, late‐onset Alzheimer's disease; DS, Down syndrome; DSAD, Down syndrome with Alzheimer's disease; MC, middle‐aged control; PMI, *post mortem* interval; YC, young control.

### 2.2 Case selection

Cases were selected to form six groups: DS without Alzheimer's disease neuropathology, Down syndrome with Alzheimer's disease (DSAD) with both AD neuropathology and a clinical consensus diagnosis of dementia, late‐onset AD with AD neuropathology and a clinical consensus diagnosis of dementia, young controls (YC) without AD neuropathology, middle‐aged controls (MC) without AD neuropathology, and aged controls (AC) without Alzheimer's disease neuropathology and a clinical consensus diagnosis of nondemented. As cases in the DS group (< 40 years) were limited, we first identified all available cases from the NIH Neurobiobank under the age of 40 years and matched cases based on age and sex to YC. Next, cases within the DSAD group were selected to include individuals with a clinical consensus diagnosis of dementia in addition to AD neuropathology and to exclude cases with mosaic and partial trisomy. MC cases from the NIH Neurobiobank were selected to match the average age of DSAD cases. The AC group had insufficient evidence of AD pathology, were non‐demented at the time of death, and matched the average age of the AD group, who were diagnosed with dementia and with AD neuropathology. Within each group, individuals were included to match for average PMI as well as to include equal amounts of males and females, based on sample availability.

**RESEARCH IN CONTEXT**

1. **Systematic review**: An extensive survey of literature using PubMed was conducted. Several studies report the timing of amyloid‐β (Aβ) and phosphorylated tau (p‐tau) emergence by neuropathology in the brains of people with Down syndrome (DS), as discussed in the Introduction and Discussion sections of this article.
2. **Interpretation**: Our findings from a large cohort of *post mortem* brain tissue provide a framework for the timing of AD development based on neuropathological signatures of Aβ and p‐tau as a function of age and sex in individuals with DS. This study expands upon previous literature that the progression of AD in DS follows a similar staging as in late‐onset AD, as well as identifies regional sex differences in this population.
3. **Future directions**: This study highlights a critical age for neuropathology development in individuals with DS and suggests women with DS may have a faster progression, given differences seen in the occipital cortex. Future investigation would benefit from focused examinations of regional AD neuropathology in longitudinal neuroimaging and fluid biomarkers. Understanding the timing at which key biomarkers emerge with age and sex based on neuropathology will inform the design and interpretation of clinical trials for people with DS.

### 2.3 Immunohistochemistry

Tissue was sectioned at 30 µm using a vibratome. Sections were stored at 4°C in phosphate buffered saline (PBS) with 0.02% sodium azide. Immunohistochemistry was performed on individual free‐floating sections. Serial sections were stained for phosphorylated tau (AT8, Invitrogen, MN1020) and amyloid‐β (6E10, Biolegend, #803003) (Table S1; Figure S1A, B). Sections were randomized such that the experimenter was blind to groups. Sections were washed in Tris‐buffered saline (TBS), then endogenous peroxidases were blocked using 1% hydrogen peroxide and 3% methanol for 30 min. Antigen retrieval was performed using sodium citrate buffer (pH 6) for 30 min in 80°C water bath followed by 90% formic acid for 5 min for the Aβ staining. Following antigen retrieval in 30% bovine serum albumin (BSA) for 1 h, tissue was incubated in primary antibodies overnight at 4°C. After washing in TBS with Triton‐X three times for 5 min, a 1‐h incubation with secondary antibody (anti‐mouse IgG, Vector, BA‐2000) at room temperature was used (Table S1). Additional washes with TBS and Triton‐X occurred prior to incubation with ABC reagent for 1 h at room temperature. 3,3′‐diaminobenzidine (DAB) was added to sections and incubated for 7 min for visualization of the stain. Sections were counterstained in cresyl violet and then dehydrated using increasing concentrations of ethanol. Dibutylphthalate polystyrene xylene (DPX) mounting media was used for cover slipping. Slides were allowed to dry for at least 24 h before imaging.

### 2.4 WSI analysis

**FIGURE 1:**
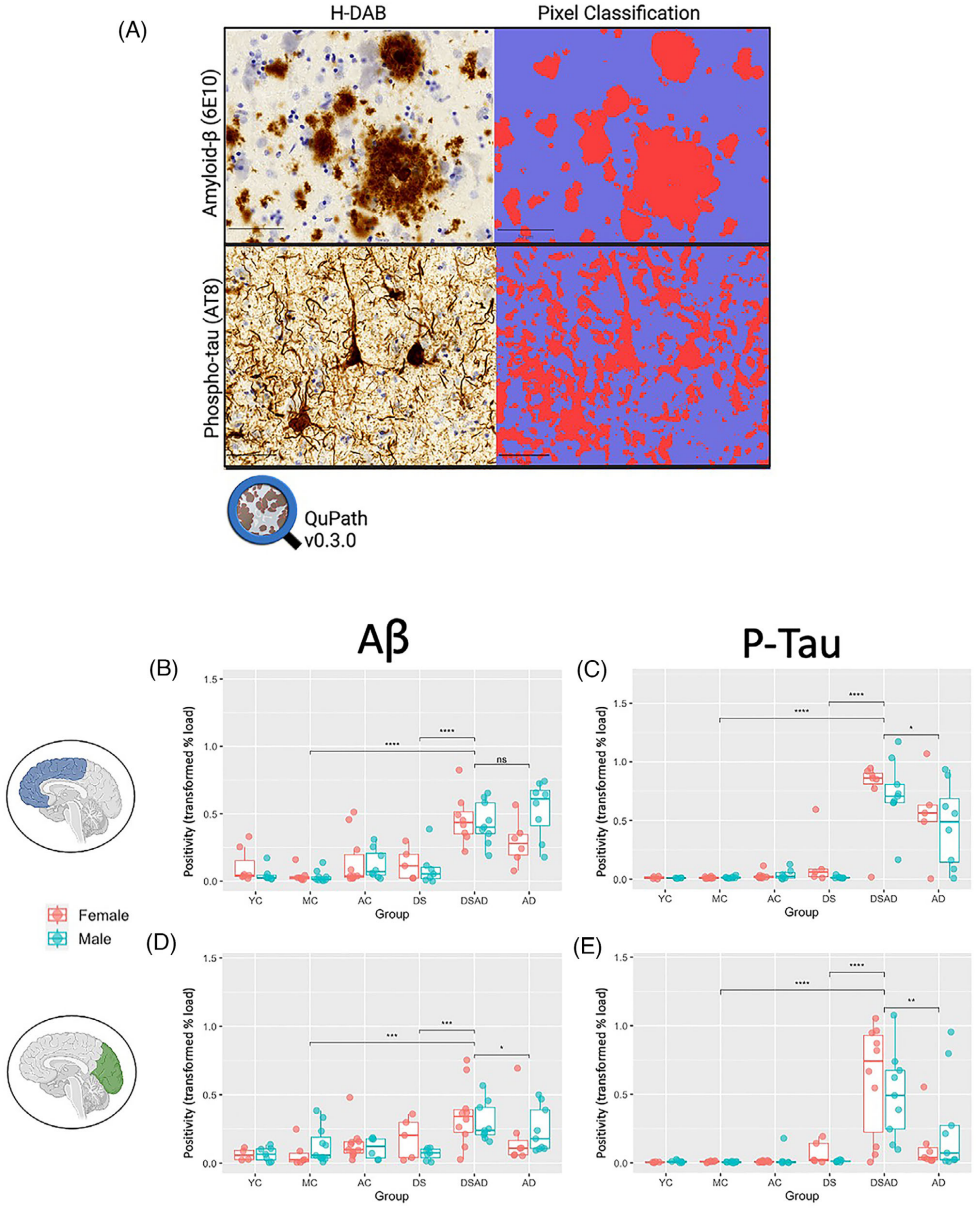
Aβ and p‐tau are significantly elevated in DSAD compared to late‐onset AD and age‐matched controls. (A) Representative images show immunohistochemical labeling of Aβ (6E10) and p‐tau (AT8) in fixed human *post mortem* tissue. Right side of the grid shows an overlay of a trained pixel classifier in Qupath (Random Trees). (B,C) Differences in the level of Aβ and p‐tau in the frontal cortex. (D,E) Differences in the level of Aβ and p‐tau in the occipital cortex. The Mann–Whitney *U* test was used to make specific group comparisons and to compare females and males with DSAD. *indicates *p* < 0.05, **indicates *p* < 0.01, *** indicates *p* < 0.001, **** indicates *p* < 0.0001. Aβ, amyloid‐β; DSAD, Down syndrome with Alzheimer's disease; AD, Alzheimer's disease p‐tau, phosphorylated tau

Mounted sections were scanned using the Leica Versa Aperio Scanner. Whole slide images (WSI) were analyzed using QuPath software (version #0.3.0), see Figure 1A.^23^ Training images were randomly selected from cases with a range of positive or negative labeling (both higher and lower background). A pixel classifier based on Random Trees, which is a more generalizable model requiring fewer parameters for performance, was trained based on annotations of positive staining. Annotations included staining representative of both the FCX and OCC regions, to ensure generalizability. Performance was assessed visually on a random area of tissue with positive staining that was not used in the training image. A blinded observer placed annotation boxes (600 × 600 µm) at random, with five boxes placed in gray matter. A trained pixel classifier was used to quantify positive staining based on the optical density of the DAB stain. Raw positive area was then divided by the total area to provide a load value in the assessed area, which we refer to as “positivity” throughout the study. Training images were created for Aβ and p‐tau, and positive and negative annotations were added by an unbiased observer until pixel classifier performance was comparable to manual annotations (> 90%) (Figure 1A). The trained pixel classifier was then applied to annotation boxes across all cases (Figure S2).

### 2.5 Statistical analyses

All data analysis and plot generation were conducted in R software (v4.2.2). Data were tested for normality using the Shapiro–Wilk test. The impact of PMI on staining intensity was examined through correlations with p‐tau and Aβ positivity. A student's *t*‐test or Mann–Whitney *U* test was used to compare men and women on the severity of pathological markers. The Kruskal–Wallis test was used to make diagnostic group comparisons in age, PMI, and protein loads. Spearman rank correlations using arc‐sin transformed positivity data were calculated. Segmented linear regression estimated inflection points and tested for significance using the Davies test. In addition, the Pettitt test estimated breakpoints using a rank‐based nonparametric analysis. A bootstrapping analysis for both the segmented regression and nonparametric models was conducted to support breakpoint estimates in frontal and occipital cortices for Aβ and tau. A total of 1000 bootstrap resamples were generated, with each sample randomly drawn with replacement from the original dataset. Mean positivity data were modeled using beta binomial regression to estimate the rate at which each cortex shows Aβ and p‐tau positivity.

## 3 RESULTS

### 3.1 Sample

This study included 156 cases, 36 of which had both frontal and occipital cortices available. For the frontal cortex region, we included cases as follows: 14 DS (6F/8 M) (< 40 years no AD pathology; DS), 18 DSAD (9F/9M) (> 40 years with AD pathology; DSAD), 15 late‐onset AD cases (6F/9M) (> 65 years with AD pathology; AD) and 50 control cases (no AD pathology) (Table 1). For the occipital region, we included cases as follows: 13 DS (5F/8M), 19 DSAD (10F/9M), 16 AD (7F/9M), and 47 control cases (Table 1). Apolipoprotein E (APOE) data were available for 21 people from the DSAD cohort; there were 10 APOE ε4 carriers and 11 non‐APOE ε4 carriers. Within the DS/DSAD groups, the distributions of race and ethnicity are approximately: 80% non‐Hispanic white, 13% African American, 4% Hispanic/Latino, and 3% Other (Asian, Native Hawaiian and Pacific Islander, and not stated/unknown). Although the groups differed in PMI, there was no correlation of PMI with Aβ and p‐tau.

### 3.2 Comparison of DSAD and AD

There were differences in Aβ and p‐tau loads in both OCC (Kruskal–Wallis; Aβ: *χ* ^2^(5) = 32.54, *p* < 0.0001, *χ* ^2^(5) = 61.29, p‐tau: *p* < 0.0001) and FCX (Kruskal–Wallis; Aβ: *χ* ^2^(5) = 56.11, *p* < 0.0001; p‐tau: *χ* ^2^(5) = 46.83, *p* < 0.0001) across diagnostic groups. These differences were driven by higher loads in the DSAD and AD groups for Aβ (FCX: *p* < 0.0001, OCC: *p* < 0.001) and p‐tau (FCX: *p* < 0.0001, OCC: *p* < 0.0001) compared to controls. FCX Aβ in the DSAD and AD groups were similar, but p‐tau levels were higher in the frontal cortex of people with DSAD compared with those with AD (Figure 1B–C). The DSAD group had higher Aβ and p‐tau loads in the occipital cortex compared to AD cases (*p* < 0.05) (Figure 1D–E). Compared to the DS group, the DSAD group had higher Aβ (FCX: *p* < 0.0001, OCC: *p* < 0.001) and p‐tau (FCX: *p* < 0.0001, OCC: *p* < 0.0001) (Figure 1B–E).

### 3.3 Age effect

**FIGURE 2:**
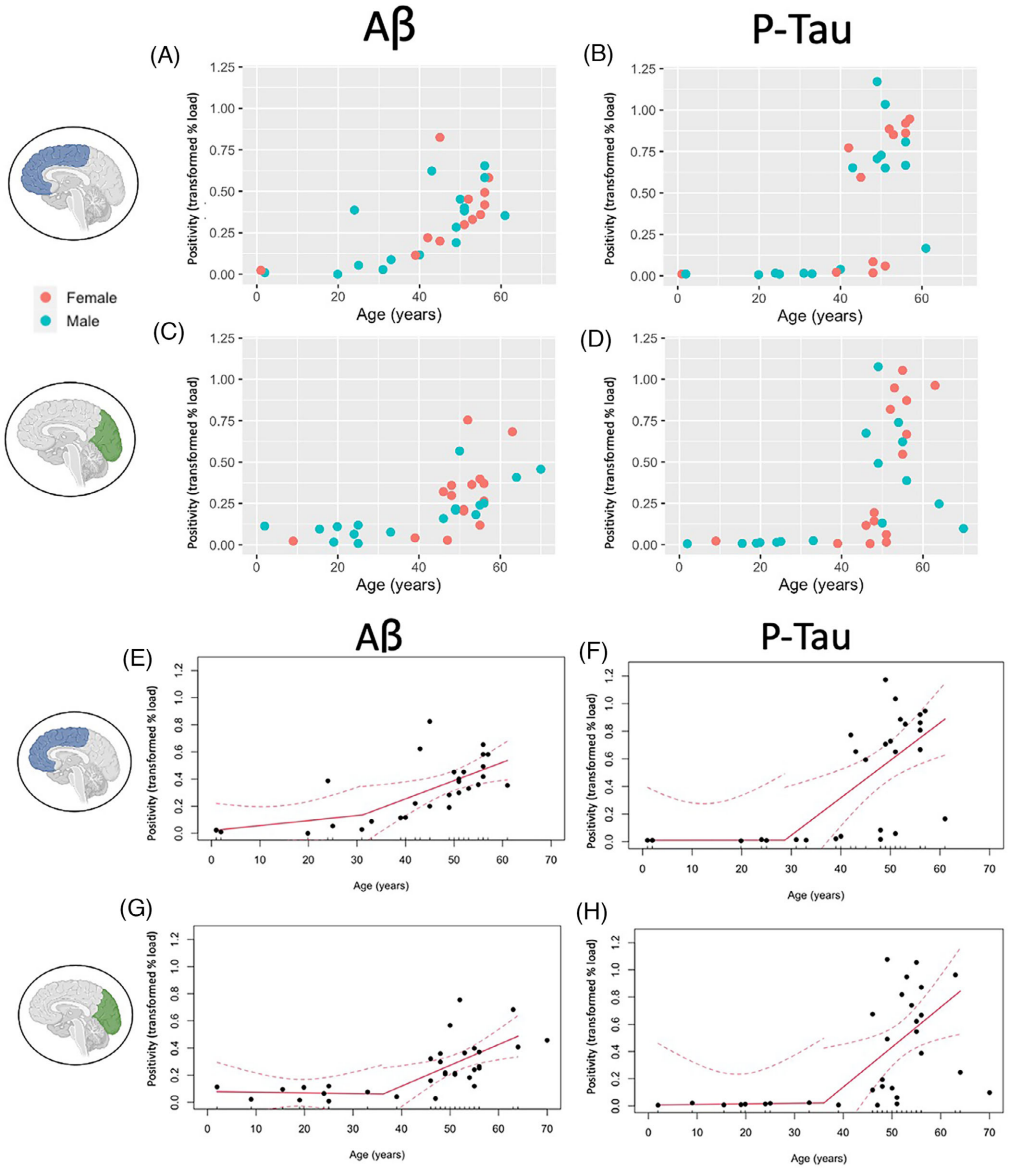
Pathology in DS and DSAD is significantly elevated after the age of 40. (A–D) Aβ and p‐tau are highly correlated with age in the DS population. All markers were significantly positively correlated with age (*p* < 0.001). (E–H) Segmented linear regression was used to determine inflection points in both frontal and occipital cortices for Aβ and p‐tau. Dotted lines indicate 95% confidence intervals. Aβ, amyloid‐β; DS, Down syndrome; DSAD, Down syndrome with Alzheimer's disease; p‐tau, phosphorylated tau

We plotted Aβ and p‐tau load as a function of age in DS and DSAD. Aβ (Figure 2A) and p‐tau (Figure 2B) increased with age in the FCX and OCC (Figure 2C and D). Because amyloid deposition is hypothesized to accelerate tau deposition, we plotted the association of p‐tau levels across age and represented levels of Aβ as point color in the same cases (Figure S3). We confirmed that cases with higher levels of p‐tau tended to have elevated levels of Aβ as well.

Given the stronger association of age with Aβ and p‐tau after 35 years of age, we calculated inflection points in both markers for FCX and OCC. Using piecewise linear regression, we estimated the age at which a slope change was observed in each brain region for each pathology. Aβ in the FCX showed an inflection at 31.2 years of age (Figure 2E) with p‐tau at 28.7 years of age (Figure 2F). In the OCC Aβ inflected at age 36.3 years of age (Figure 2G) and p‐tau at 35 years of age (Figure 2H). The Davies test was used to determine if the slopes before and after the inflection point differed significantly. In our analyses we did not find significance by the Davies test, but we report the estimated inflection point from the piecewise linear regression. The calculated inflection points are shown in Table 2. We note that the case with DSAD who was 70 years at death appeared to be an outlier in the OCC and, thus, provide estimates without this case included.

**TABLE 2:** Estimated age inflection points and bootstrap analysis of Aβ and p‐tau positivity

Estimated inflection points									
	Frontal cortex		Occipital cortex						
	Estimate (Age, years)	Standard error	Estimate (age, years)	Standard error					
Aβ	31.188	15.047	36.251^a^	8.894^a^					
p‐tau	28.678	10.999	35.998^a^	10.491^a^					

Bootstrap analysis results									
	Frontal cortex					Occipital cortex			
	Parameter	Estimate	Lower CI	Upper CI		Parameter	Estimate	Lower CI	Upper CI
Aβ	Breakpoint	37.94726596	9.51929981	56.9999000	Aβ	Breakpoint	40.370400146	19.779326238	56.011609202
	Slope before breakpoint	0.00157099	−0.01376789	0.0138199		Slope before breakpoint	0.001126254	−0.005852411	0.009829939
	Slope after the breakpoint	0.01820299	−0.03635961	0.1284311		Slope after breakpoint	0.019569677	−0.027848433	0.056833990
p‐Tau	Breakpoint	42.565157229	23.2837778024	56.99990000	P‐tau	Breakpoint	40.370400146	19.779326238	56.011609202
	Slope before breakpoint	0.008009275	−0.0002040052	0.02560063		Slope before breakpoint	0.001126254	−0.005852411	0.009829939
	Slope after the breakpoint	−0.032814366	−0.1689075062	0.07220677		Slope after breakpoint	0.019569677	−0.027848433	0.056833990

Abbreviations: Aβ, amyloid‐β; p‐tau, phosphorylated tau.

^a^ Indicates the case aged 70 was removed from analysis. Datapoint is still shown in the graph (Figure 2G, H).

We performed a bootstrapping analysis to resample our data and examined the confidence intervals around the inflection point and the slopes before and after resampling (Table 2). Wide confidence intervals surrounding the inflection points after bootstrapping suggest variability in our estimate of an age‐related inflection point in pathology. However, narrow confidence intervals surrounding the slopes before and after the inflection points suggest a more reliable estimate of inflection.

In the case of p‐tau, the steep increase observed may represent a two‐group distribution. Thus, we implemented a rank‐based analysis that does not assume linearity to investigate if we observe a different change point in p‐tau. By the Pettitt test, we observed change points at 40 and 48 years in the FCX (*p* < 0.001) and 48 years in the OCC (*p *< 0.01) (Figure S4A, C). We implemented the same bootstrap analysis as with the segmented linear models after obtaining the initial estimates and reported confidence intervals (percentile method). From 1000 bootstrap replicates of the procedure, the data strongly support an estimate in the range from 19.87 to 56 years of age in the FCX and from 19 to 56 years of age in the OCC, each with a 95% level of confidence (Figure S4B, D; Table S2). The inflection points from the initial piecewise linear regression fall within these estimates, though we note that the Pettitt test results reported later estimated change points, which may be more reflective of p‐tau's deposition trajectory.

**FIGURE 3:**
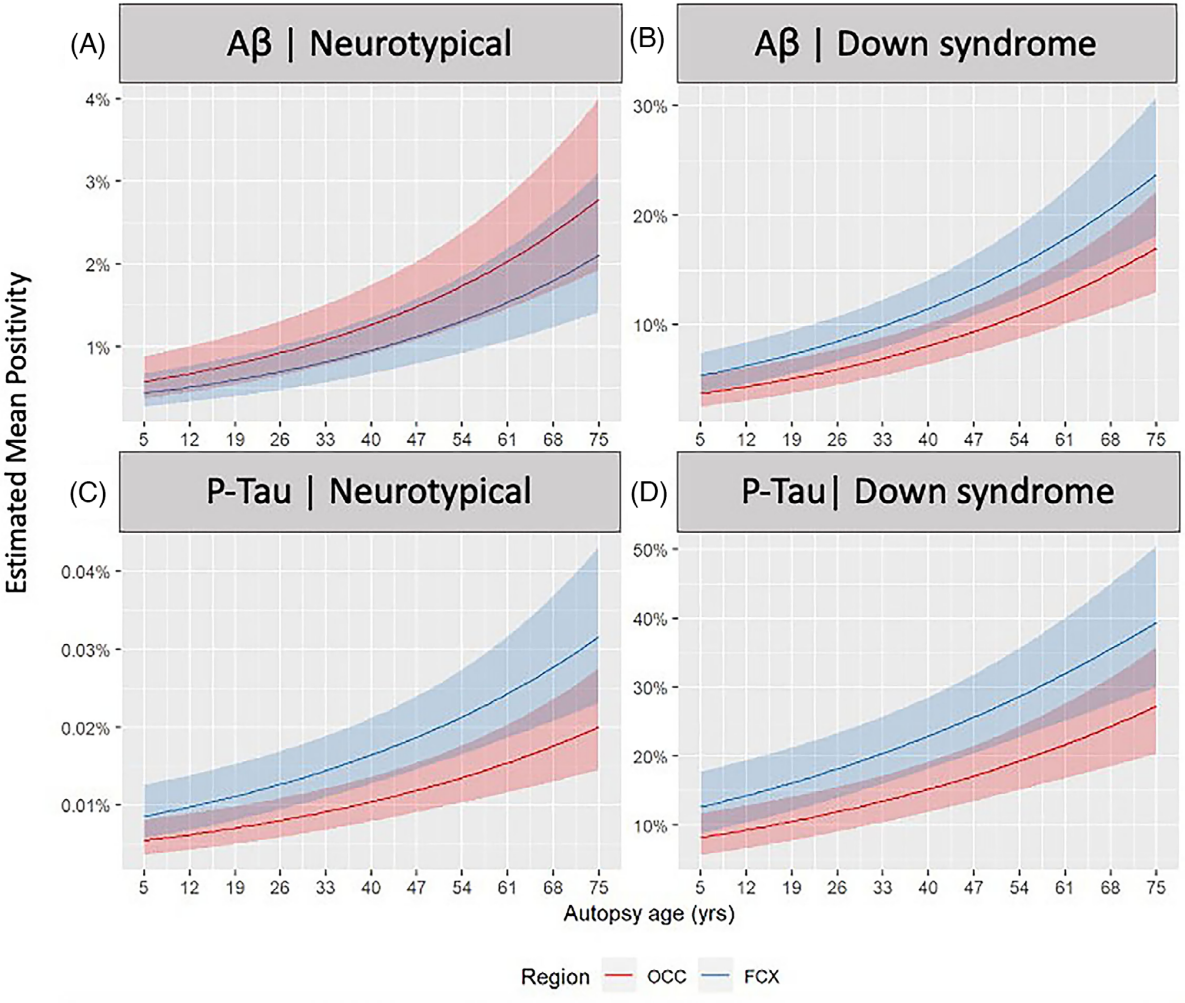
Frontal cortex shows earlier and faster estimated accumulation rates in DS and DSAD. Estimated mean positivity rate for Aβ and p‐tau by age, region, and class. Estimates based on β‐binomial regression with 80% confidence interval. Mean positivity increases in all groups with age at autopsy. Mean rates of Aβ are around 10 times higher than those of age‐matched controls. Mean p‐tau positivity was about twice as high as Aβ positivity. The rate of accumulation in the FCX appeared higher than in the OCC. Estimated coefficients in beta‐binomial regression of positivity rate on *post mortem* age, region, and diagnostic class. Aβ, amyloid‐β; DS, Down syndrome; DSAD, Down syndrome with Alzheimer's disease; FCX, frontal cortex; OCC, occipital cortex; p‐tau, phosphorylated tau

We next investigated the rate of Aβ and p‐tau accumulation using a regression that included mean positivity to estimate the pathologic deposition in the two different cortical regions, given a participant's age. Using β‐binomial regression analyses, we estimated the progression of Aβ and p‐tau in FCX and OCC. Overall, mean positivity increased in both DSAD and controls with age, but estimates for positivity in controls remained below 4% coverage for Aβ and less than 1% coverage of p‐tau. In the DS and DSAD groups, the frontal cortex showed consistently earlier and elevated pathology than the occipital cortex by our estimates (Figure 3, Table 3, Figure S5).

**TABLE 3:** Estimated coefficients in beta‐binomial regression of positivity rate on *post mortem* age, region, and diagnostic class

Model		Estimate		80% CI			
Parameter	Term	Estimate	SE	Lower	Upper	*p*‐Value	df
**Amyloid‐β**							
Mean	Ctrl (OCC, 40 pm yr)	−4.364	0.253	−4.691	−4.038	< 0.001	111
	Age (pm yr)	0.023	0.005	0.016	0.03	< 0.001	111
	DS (in OCC)	1.889	0.309	1.49	2.288	< 0.001	111
	FCX in Control	−0.283	0.25	−0.606	0.04	0.13031	111
	FCX in DS	0.655	0.334	0.224	1.086	0.02624	111
Scale	Control	−3.28	0.27	−3.628	−2.931	< 0.001	111
	DS	1.35	0.347	0.903	1.797	< 0.001	111
**p‐tau**							
Mean	Control (OCC, 40 pm yr)	−9.168	0.212	−9.441	−8.895	< 0.001	101
	Age (pm yr)	0.019	0.005	0.012	0.026	< 0.001	101
	DS	7.367	0.252	7.042	7.692	< 0.001	101
	FCX	0.456	0.185	0.217	0.694	0.00769	101
Scale	Control	−8.336	0.25	−8.657	−8.014	< 0.001	101
	DS	7.935	0.32	7.522	8.347	< 0.001	101

*Note*: Control denotes the neurotypical controls. Age (pm yr) denotes *post mortem* age in years, DS an indicator of Down syndrome, and FCX an indicator of the frontal versus occipital cortex (OCC). FCX in DS denotes the modification of the regional association by DS. Degrees‐of‐freedom (df) denotes the residual or error degrees of freedom in the fitted model. Standard errors (SE) estimate the precision or accuracy of the estimated parameter. *p*‐values are directional, looking left and right, as the sign of the coefficient.

Abbreviation: CI, confidence interval.

### 3.4 Sex effects

Sex is associated with AD neuropathology in the general population but has not been well characterized in DS. Within the DS group, women had higher levels of p‐tau in the FCX compared to men (*p* < 0.05, effect size = 0.5895), but similar levels of Aβ (*p* > 0.05). Within the OCC, men and women did not differ Aβ or p‐tau load. However, women with DSAD on average had higher levels of p‐tau than men with DSAD, despite having similar levels of Aβ (Figure 1C, E).

**FIGURE 4:**
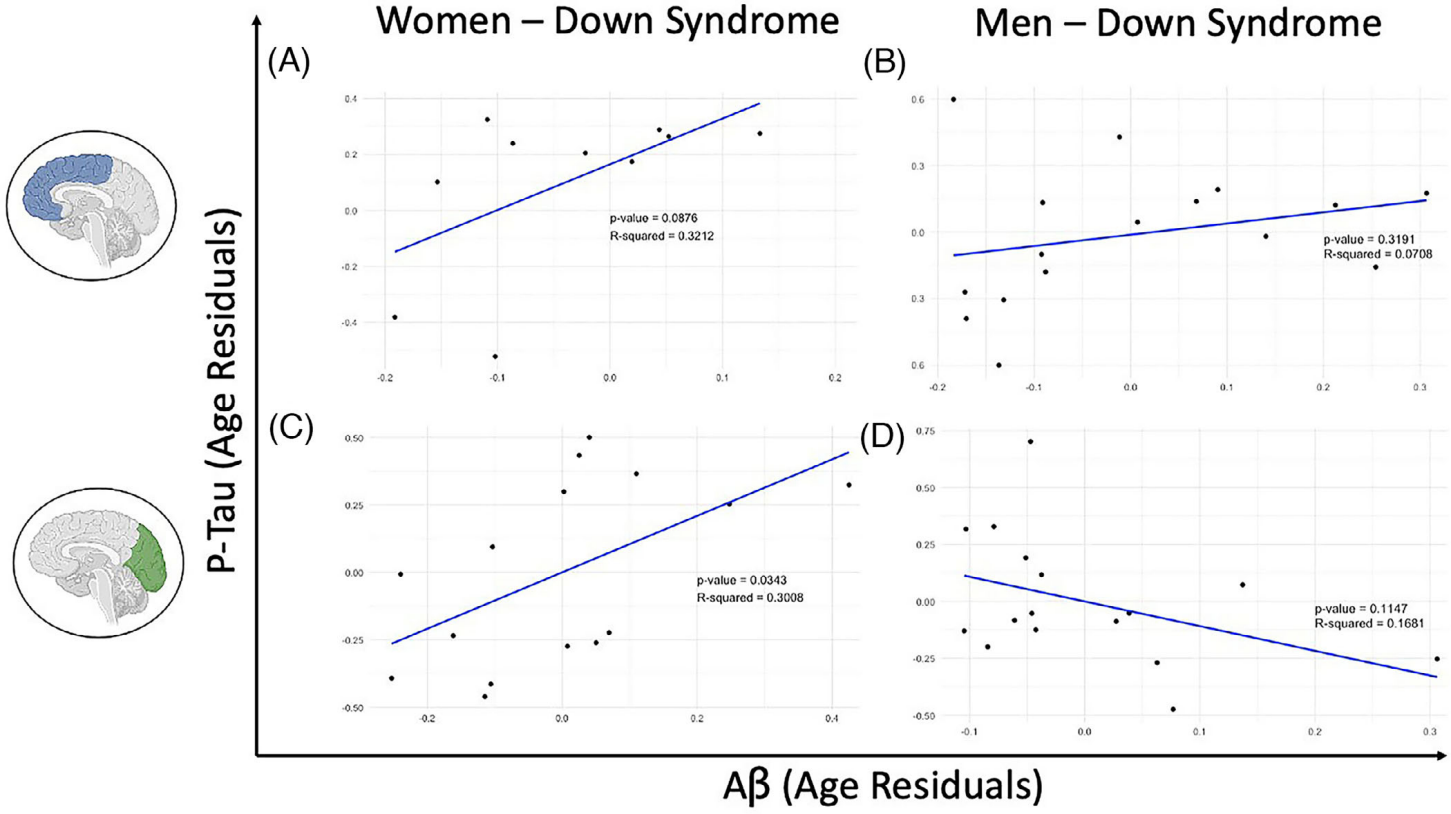
Aβ and p‐tau are significantly associated in the occipital cortex of women with DS. Partial correlation scatter plots of Aβ and p‐tau (residuals from regression on age), in men and women with DS and DSAD, show no significance in the frontal cortex (*p* > 0.05). Aβ and p‐tau show no significant association in men in the occipital cortex but demonstrate significance in the occipital cortex of women (*p* < 0.05). Aβ, amyloid‐β; DS, Down syndrome; DSAD, Down syndrome with Alzheimer's disease; p‐tau, phosphorylated tau.

The correlation between age and p‐tau in the OCC was stronger in women than in men (Figure 4). To further investigate whether the OCC is particularly affected in women, we examined correlations between Aβ and p‐tau after adjusting for age. We hypothesized that women would show a greater association between Aβ and p‐tau, suggesting a more advanced stage of pathology. In the frontal cortex, there was a positive correlation between Aβ and p‐tau (*R* ^2^ = 0.3212, *p* = 0.0876) in women (Figure 4A), but not in men (*R* ^2^ = 0.0708, *p* = 0.3191) (Figure 4B). Similarly, in the occipital cortex, there was a strong positive correlation between Aβ and p‐tau in women (*R* ^2^ = 0.3008, *p* = 0.0343) (Figure 4C), but not in men (*R* ^2^ = 0.1681, *p* = 0.1147) (Figure 4D).

## 4 DISCUSSION

We examined the association of age and sex with AD pathology in DS. We observed Aβ deposition as young as 10 years, though mature plaques were not observed until over the age of 30 years. Tau deposition was not detected until after the age of 40 years in DS. We estimated age inflection points within the DS cohort and observed very little gap in age between the timing of Aβ and p‐tau accumulation within each brain region. However, across brain regions, FCX AD pathology occurs prior to OCC pathology. Specifically, in the OCC, the age inflection point of Aβ was approximately 5 years after the FCX, with p‐tau pathology present ∼7 years later, on average. Thus, Aβ and p‐tau pathology may co‐evolve within each brain region, and FCX is affected 5–7 years prior to OCC. In implementing a rank‐based approach to the p‐tau data, we observed later change points at about 40 years in FCX and 48 years in OCC. Given that elevated levels of p‐tau were only observed after the age of 40, these estimates could indicate a critical age at which p‐tau pathology rapidly develops. We observed higher levels of p‐tau but not Aβ pathology in both the frontal and occipital cortex of women with DS compared with men with DS, although these differences were not reliable. However, there may be faster progression of AD neuropathology in women with DS, given that Aβ and p‐tau pathology exhibited a strong positive correlation in women with DS after adjusting for age in the OCC, with more subtle sex differences seen in higher levels of p‐tau in women, suggesting a more advanced stage of pathology.

Our findings of early deposition of Aβ in DS is consistent with previous observations in individuals as young as 1 year,^24^ 8 years,^25^ and 12 years.^10^ We found the FCX develops Aβ pathology at ∼31 years of age while Aβ in OCC develops after 36 years of age, thus there is a 4‐ to 5‐year progression. The pattern of FCX showing Aβ earlier than OCC aligns with Aβ Thal phases and neuroimaging studies from late‐onset AD.^26^, ^27^ P‐tau similarly accumulates in the FCX beginning after ∼28 years of age, with critical changes estimated ∼40 years of age. Whereas OCC shows p‐tau at ∼36 years of age, with a critical change point estimated at ∼48 years, suggesting a longer gap of at least 7 years. This pattern is consistent with Braak tangle staging.^20^, ^28^ It was also not surprising that within each brain region, that Aβ and p‐tau appeared to accumulate relatively close together in age as amyloid and p‐tau PET studies in DS also describe a narrow window of 2.5–5 years between these two events.^21^

We hypothesized that women with DS would have higher p‐tau levels but similar Aβ levels to men with DS. The outcomes of this study suggest that sex differences are brain region‐specific. Sex differences in AD pathology in DS were largest in the OCC, which accumulates Aβ and p‐tau later with age and disease. Women with DSAD had higher levels of p‐tau pathology in the OCC than men, suggesting a more rapid progression of p‐tau pathology as AD evolves. This observation agrees with a previous report where women with DSAD had more severe neocortical NFT at autopsy, as well as findings from the neurotypical population suggesting women are more vulnerable to p‐tau accumulation, even prior to dementia.^11^, ^13^, ^19^ These observations suggest that genetic, hormonal, vascular, and/or social determinants that are patterned by sex or gender may contribute to the severity of tau pathology

Several studies implicate a more posterior distribution of cerebrovascular disease in AD.^22^, ^29^ Women with DS had higher white matter hyperintensity volume on MRI, a marker of small vessel cerebrovascular disease, compared with men, particularly in posterior regions.^30^ Thus, one possible reason for higher p‐tau levels in the OCC in women with DS may be linked to cerebrovascular pathology, but with an unclear mechanism.

Despite observed differences, very few reports indicate clinical differences between men and women with DSAD. Women with DS show similar age of onset of dementia, which may point to greater capacity for cognitive reserve if women do have more advanced stages of pathology.^31^ Findings from autosomal dominant AD (ADAD) indicate women have greater working memory and verbal comprehension, yet higher levels of plasma neurofilament light (NfL), a marker for neurodegeneration, further underscoring the potential for resilience.^32^, ^33^ No sex differences were identified in amyloid or tau PET in ADAD, though it has not been extensively investigated.^34^ The potential for resilience in women was investigated in the neurotypical population as well, with several studies reporting women may have better compensatory mechanisms (which may reflect resilience) for pathologic burden, despite having more severe AD pathology loads at autopsy.^11^, ^35^ In a recent report of 260 adults with DS, women showed higher plasma total p‐tau levels compared to men yet performed better on the modified Cued Recall Test.^36^ These findings suggest that while women may be more at risk for developing pathology, they may exhibit more cognitive reserve than men or women outperform men on tests of memory in general. In addition, sex differences in transcriptomic profiles of individuals with DSAD showed that women have elevated transcripts related to inflammation and oxidative stress, further underscoring that women may have different pathways for responding to AD pathology.^37^

Many authors suggest that menopause and hormones may explain sex differences in AD. There is significant evidence from the general population suggesting that menopause and hormone therapy use mediate the relationship between Aβ and p‐tau in cognitively unimpaired women.^38^ In women with DS, earlier age at menopause correlated with earlier onset of clinical dementia.^39^, ^40^

APOE ε4 is a known risk factor for AD, particularly in women.^41^ This possibility may also hold true for people with AD; age at diagnosis is earlier in women with DS who are APOE ε4 carriers, but not in men with DS.^31^ Women who were APOE ε4 carriers had lower cerebrospinal fluid (CSF) Aβ42/40 ratios and lower hippocampal volumes compared to women without the ε4 allele.^31^ The current study was underpowered to evaluate the role of APOE ε4 on the higher p‐tau loads observed in the OCC in women with DS. Future investigations will benefit from the inclusion of additional cases and brain regions with APOE and menopause information.

The current study has some additional limitations. It is important to consider that these data are necessarily cross‐sectional and represent a single time point of pathology. In addition to our calculation of age inflection points for Aβ and p‐tau across the FCX and OCC, it will be critical to expand studies in the 30‐ to 40‐years age range to understand mechanisms leading to rapid accumulation. However, it is also important to note that previous neuroimaging studies observed variable onset of amyloid deposition, but fairly rapid and consistent p‐tau deposition and dementia onset once there is evidence of amyloid pathology.^42^ Our findings of a short interval between Aβ and p‐tau deposition in FCX and OCC cortices are consistent with these previous observations. We also note that NIH Neurobiobank cases have limited clinical information and may have also had contributing comorbidities. We are also limited by the ethnicity of our sample, as predominantly non‐Hispanic White populations are represented. In the future, we hope increased recruitment and community outreach can begin to close this gap in our population.

With new AD treatments currently available and several others in development, it is important to understand the natural history of AD progression and how it differs between men and women to inform detection thresholds and treatment plans. In reports of lecanemab, a recently United States Food and Drug Administraion (FDA)‐approved AD therapeutic, women benefited less from treatment than men.^43^ We anticipate this difference will be apparent in the DSAD population as well, and it will be critical to consider sex differences explicitly in the design and interpretation of AD clinical trials in DS.

## CONFLICT OF INTEREST STATEMENT

The authors declare no conflicts of interest. Author disclosures are available in the Supporting Information.

## CONSENT STATEMENT

All studies were in de‐identified human brain tissue that did not meet the definition of human subjects research. Consent was not necessary.

## APPENDIX—COLLABORATORS

Investigators: Howard J. Aizenstein, MD PhD; Beau M. Ances, MD PhD; Howard F. Andrews, PhD; Karen Bell, MD; Rasmus M. Birn, PhD; Adam M. Brickman, PhD; Peter Bulova, MD; Amrita Cheema, PhD; Kewei Chen, PhD; Bradley T. Christian, PhD; Isabel Clare, PhD; Lorraine Clark, PhD; Ann D. Cohen, PhD; John N. Constantino, MD; Eric W. Doran, MS; Anne Fagan, PhD; Eleanor Feingold, PhD; Tatiana M. Foroud, PhD; Benjamin L. Handen, PhD; Jordan Harp, PhD; Sigan L. Hartley, PhD; Elizabeth Head, PhD; Rachel Henson, MS; Christy Hom, PhD; Lawrence Honig, MD; Milos D. Ikonomovic, MD; Sterling C Johnson, PhD; Courtney Jordan, RN; M. Ilyas Kamboh, PhD; David Keator, PhD; William E. Klunk, MD PhD; Julia K. Kofler, MD; William Charles Kreisl, MD; Sharon J. Krinsky‐McHale, PhD; Florence Lai, MD; Patrick Lao, PhD; Charles Laymon, PhD; Joseph Hyungwoo Lee, PhD; Ira T. Lott, MD; Victoria Lupson, PhD; Mark Mapstone, PhD; Chester A. Mathis, PhD; Davneet Singh Minhas, PhD; Neelesh Nadkarni, MD; Sid O'Bryant, PhD; Melissa Parisi, MD PhD; Deborah Pang, MPH; Melissa Petersen, PhD; Julie C. Price, PhD; Margaret Pulsifer, PhD; Michael S. Rafii, MD PhD, Eric Reiman, MD; Batool Rizvi, MS; Herminia Diana Rosas, MD; Laurie Ryan, PhD; Frederick Schmitt, PhD; Nicole Schupf, PhD; Wayne P. Silverman, PhD; Dana L. Tudorascu, PhD; Rameshwari Tumuluru, MD; Benjamin Tycko, MD PhD; Badri Varadarajan, PhD; Desiree A. White, PhD; Michael A. Yassa, PhD; Shahid Zaman, MD PhD; Fan Zhang, PhD.

## Supporting information



Supporting Information

Supporting Information
