# Correction to “Age and sex are associated with Alzheimer's disease neuropathology in Down syndrome”

**DOI:** 10.1002/alz.70914

**Published:** 2025-11-07

Andrews EJ, Ngo PT, Pascual JR, et al. Age and sex are associated with Alzheimer's disease neuropathology in Down syndrome. *Alzheimers Dement*. 2025 Jul;21(7):e70408. doi: 10.1002/alz.70408. PMID: 40673442; PMCID: PMC12268376.

In the Acknowledgments section, we failed to list one source of funding. The last sentence should include the following: “This work was also supported by the UCI Institute for Memory Impairments and Neurological Disorders/Women's Alzheimer's Movement (UCI MIND/WAM 01‐2021) Pilot Grant.”

We apologize for this error.

