# Supplementary material for: Age and sex are associated with Alzheimer's disease neuropathology in Down syndrome
Source: Alzheimers Dement. 2025 Jul 17;21(7):e70408. doi: 10.1002/alz.70408 (PMC12268376; doi:10.1002/alz.70408)
Supplement: Supplementary file 1 — Supporting Information [file ALZ-21-e70408-s001.docx]

Supplemental Figures

Supplemental Figure 1. Aβ and p-tau labeled by immunohistochemistry in postmortem human brain tissue. A) Representative images of human postmortem brain tissue stained for Aβ1-16. Plaques in DSAD were morphologically similar to those in late-onset AD cases, although presented at much younger ages. Some amyloid labeling occurred in younger DS cases. B) Representative images of p-tau (Ser 202, Thr 205) stained with AT8. Neurofibrillary tangles were present in both AD and DSAD and were not observed in controls. Extensive p-tau pathology was observed at younger ages in DSAD compared to AD.


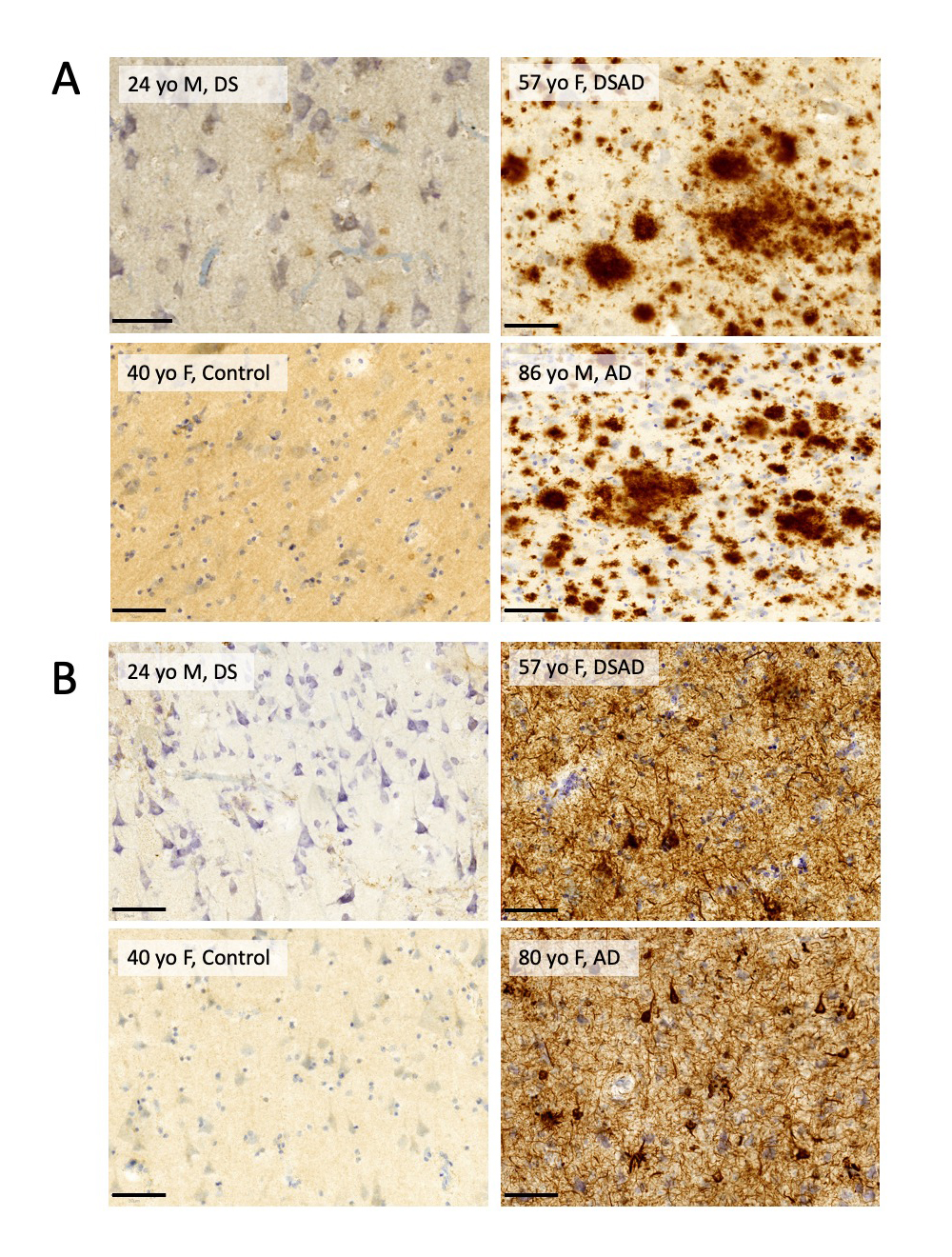


Supplemental Figure 2. Digital pathology analysis pipeline using Qupath. Steps 1-3 show representation of whole slide image acquisition. Steps 4-5 show generation of training image and preparation of pixel classifier. Steps 6-7 show example of region of interest (ROI) placement and data acquisition.


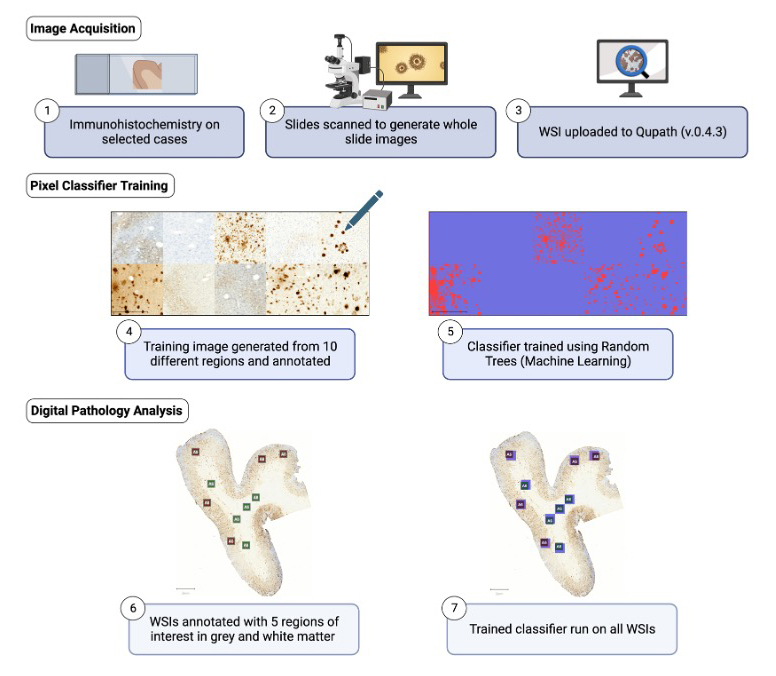


Supplemental Figure 3. Levels of p-tau by age correspond with elevated levels of Aβ. Gradient point colors represent level of Aβ positivity. Point shape indicates status group (circle: Down syndrome (DS); triangle: Down syndrome with Alzheimer’s disease (DSAD))


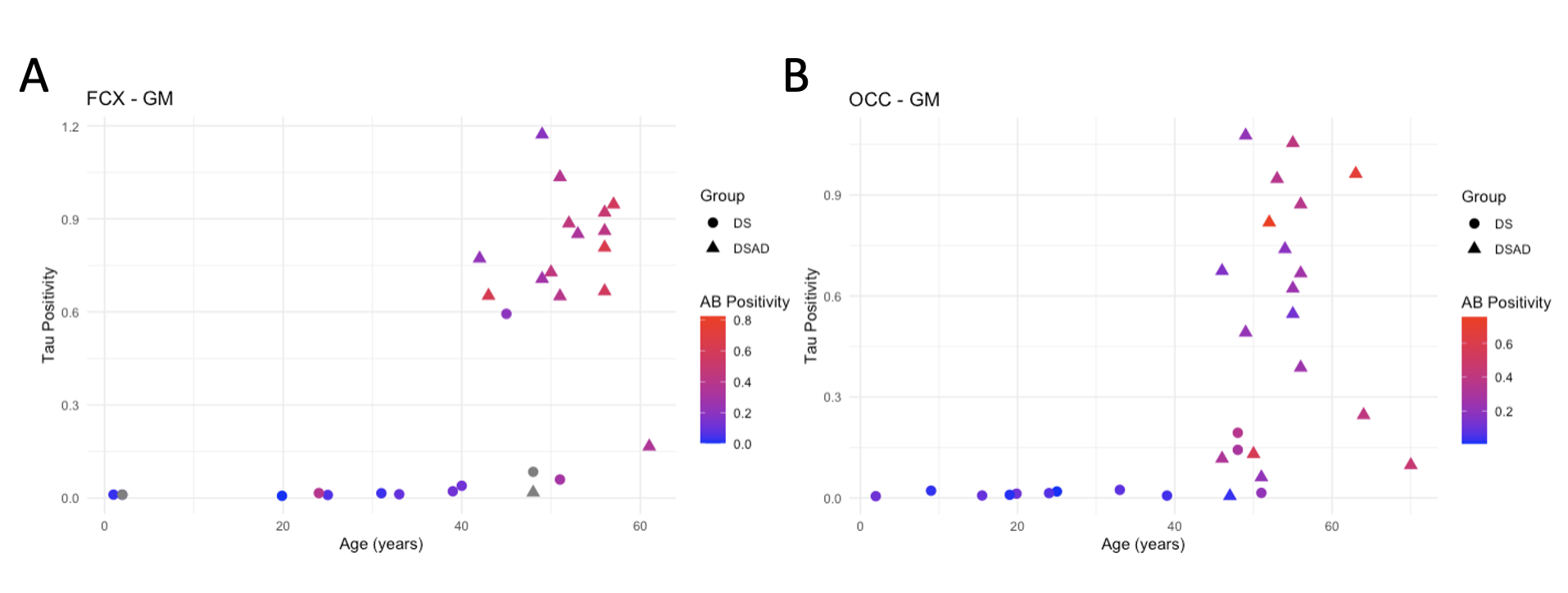


Supplemental Figure 4. Estimated changepoints of p-tau. Red line indicates estimated changepoint as detected by the Pettitt test. Bootstrap analysis was performed using the same parameters as in the previous analysis (Figure 2, Table 2). Blue lines indicate 95% confidence intervals obtained after bootstrapping analysis.


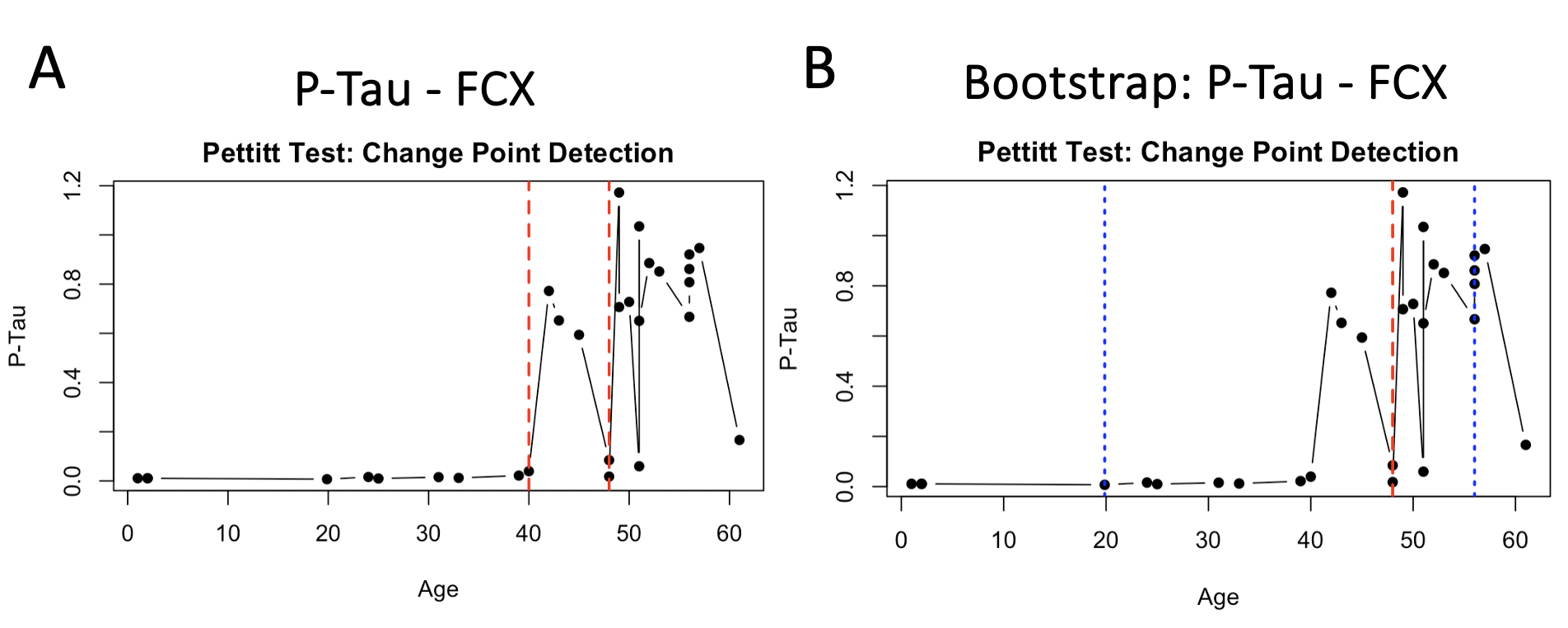


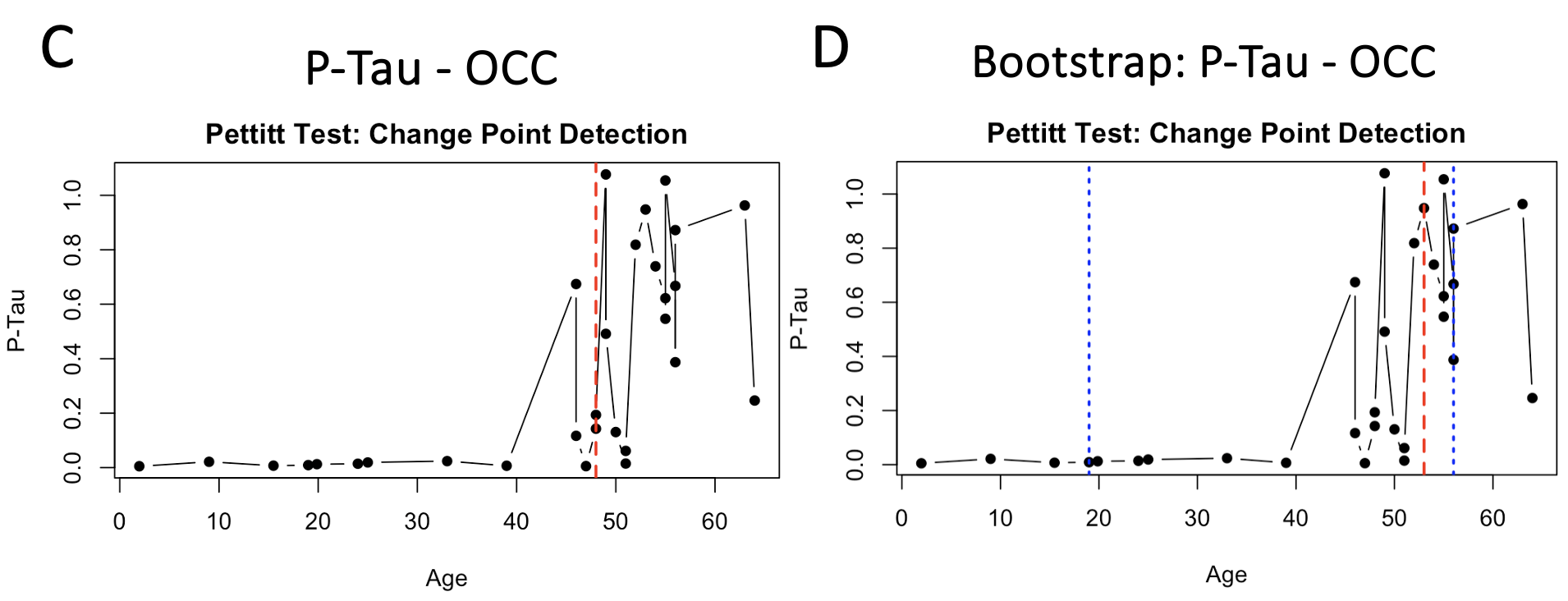


Supplemental Figure 5. The occipital lobe develops pathology later than the frontal cortex. Aβ was found to present in the frontal cortex at a 10% threshold at age 48 in FCX, followed by similar levels in the OCC at age 63. Thus, there is about a 15-year gap in Aβ development. Tau was found to reach 10% threshold at age 38 in FCX and age 59 in OCC, suggesting a 21-year gap in in p-tau development.


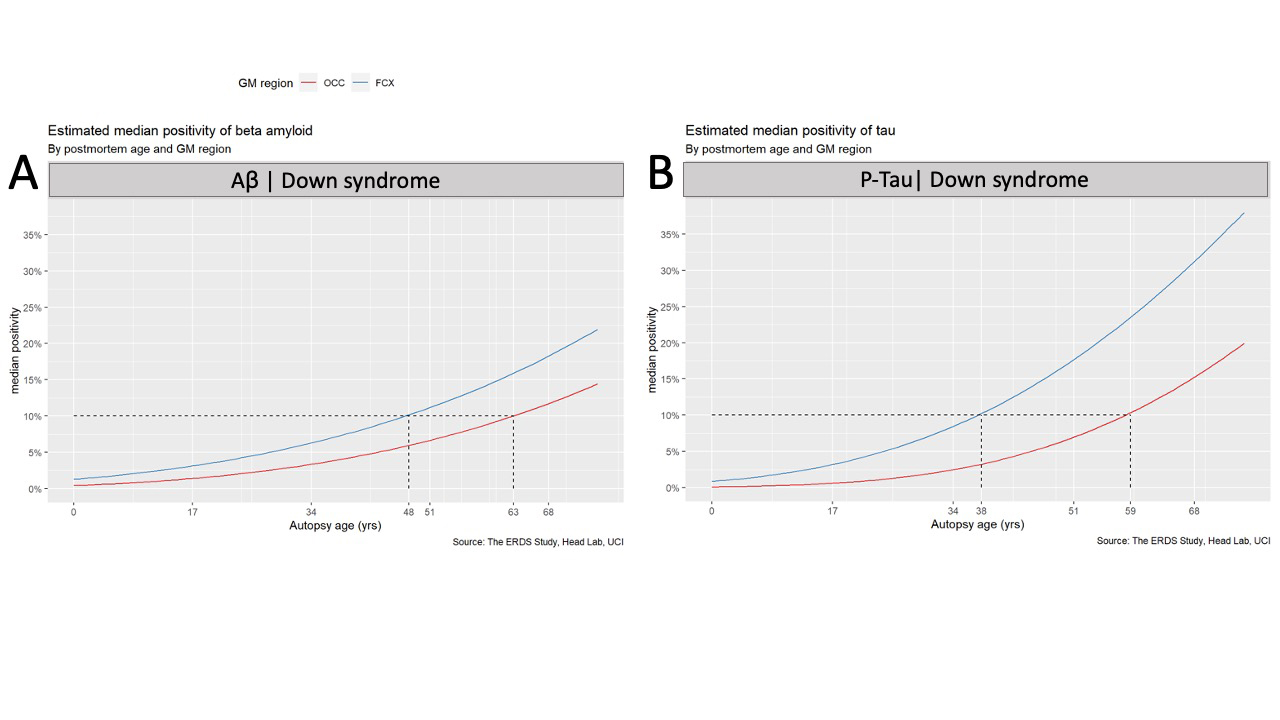


Supplemental Table 1 – Antibodies Used

| Target | Clone | Manufacturer | Catalogue Number | Lot Number | Host, Antibody Clonal Type | Working Dilution | Conditions |
| --- | --- | --- | --- | --- | --- | --- | --- |
| Amyloid Beta 1-16 | 6E10 | Biolegend | 803003 | B353947 | Mouse, monoclonal | 1:4000 | Citrate (pH 6) and 90% Formic Acid |
| Phosphorylated Tau - Serine 202, Threonine 205 | AT8 | Invitrogen | MN1020 | XD3545842 | Mouse, monoclonal | 1:3000 | None |
| Mouse IgG | - | Vector | BA-2000 | ZJ0725 | Horse, Biotinylated | 1:1000 | None |

|  | **FCX** | | | **OCC** | | |
| --- | --- | --- | --- | --- | --- | --- |
|  | **Changepoint (age, yrs)** | **U** | **P-value** | **Changepoint (age, yrs)** | **U** | **P-value** |
| **P-Tau** | 40-48 | 186 | 0.0005 | 48 | 185 | 0.0013 |

Supplemental Table 2 – Pettitt test results for p-tau

Bootstrap Results

| **Parameter** | **Estimate (age, years)** | **Lower CI (2.5%)** | | **Upper CI (97.5%)** |
| --- | --- | --- | --- | --- |
| FCX Changepoint | 48 | | 19.87 | 56 |
| OCC Changepoint | 53 | | 19 | 56 |
